# Communication and Language: Why Age Matters

**DOI:** 10.1093/geroni/igaa057.1908

**Published:** 2020-12-16

**Authors:** Alison Chasteen, Sali Tagliamonte

## Abstract

In line with this year’s 75th anniversary theme, we will show why aging matters for communication and language. Specifically, in this symposium we will show how aging affects communication and language across a variety of social contexts, social roles, and cognitive abilities. Pabst & Tagliamonte discuss the effects of aging on language use by examining an individual’s daily diary entries over 30 years, including the onset and progression of dementia. Saunders considers language and communication in the context of social interaction among persons with dementia living in a long-term care setting. Savundranayagam et al. test the efficacy of a communication intervention for personal support workers who work with persons with dementia. Chasteen & Tagliamonte consider how ageism is communicated to middle-aged and older adults in everyday life. Taken together, these presentations will provide a multidimensional lens to understanding language and communication in later life.

